# Checking Qualitative Liveness Properties of Replicated Systems with Stochastic Scheduling

**DOI:** 10.1007/978-3-030-53291-8_20

**Published:** 2020-06-16

**Authors:** Michael Blondin, Javier Esparza, Martin Helfrich, Antonín Kučera, Philipp J. Meyer

**Affiliations:** 8 Microsoft Research Lab, Redmond, WA USA; 9 University of Southern California, Los Angeles, CA USA; 10 Université de Sherbrooke, Sherbrooke, Canada; 11 Technical University of Munich, Munich, Germany; 12 Masaryk University, Brno, Czechia

**Keywords:** Parameterized verification, Liveness, Stochastic systems

## Abstract

We present a sound and complete method for the verification of qualitative liveness properties of replicated systems under stochastic scheduling. These are systems consisting of a finite-state program, executed by an unknown number of indistinguishable agents, where the next agent to make a move is determined by the result of a random experiment. We show that if a property of such a system holds, then there is always a witness in the shape of a *Presburger stage graph*: a finite graph whose nodes are Presburger-definable sets of configurations. Due to the high complexity of the verification problem (non-elementary), we introduce an incomplete procedure for the construction of Presburger stage graphs, and implement it on top of an SMT solver. The procedure makes extensive use of the theory of well-quasi-orders, and of the structural theory of Petri nets and vector addition systems. We apply our results to a set of benchmarks, in particular to a large collection of population protocols, a model of distributed computation extensively studied by the distributed computing community.

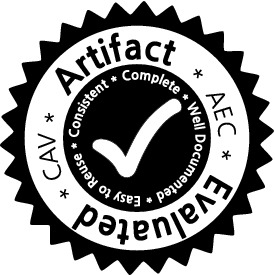



## Introduction

Replicated systems consist of a fully symmetric finite-state program executed by an unknown number of indistinguishable agents, communicating by rendez-vous or via shared variables [14, 16, 41, 46]. Examples include distributed protocols and multithreaded programs, or abstractions thereof. The communication graph of replicated systems is a clique. They are a special class of *parameterized systems*, i.e., infinite families of systems that admit a finite description in some suitable modeling language. In the case of replicated systems, the (only) parameter is the number of agents executing the program.

Verifying a replicated system amounts to proving that an infinite family of systems satisfies a given property. This is already a formidable challenge, made even harder by the fact that we want to verify liveness (more difficult than safety) against stochastic schedulers. Loosely speaking, stochastic schedulers select the set of agents that should execute the next action as the result of a random experiment. Stochastic scheduling often appears in distributed protocols, and in particular also in population protocols—a model much studied in distributed computing with applications in computational biology^1^—that supplies many of our case studies [9, 58]. Under stochastic scheduling, the semantics of a replicated system is an infinite family of finite-state Markov chains. In this work, we study *qualitative* liveness properties, stating that the infinite runs starting at configurations of the system satisfying a precondition almost surely reach and stay in configurations satisfying a postcondition. In this case, whether the property holds or not depends only on the topology of the Markov chains, and not on the concrete probabilities.

We introduce a formal model of replicated systems, based on multiset rewriting, where processes can communicate by shared variables or multiway synchronization. We present a sound and complete verification method called *Presburger stage graphs*. A Presburger stage graphs is a directed acyclic graphs with Presburger formulas as nodes. A formula represents a possibly infinite inductive set of configurations, i.e., a set of configurations closed under reachability. A node $\mathcal {S}$ (which we identify with the set of configurations it represents) has the following property: A run starting at any configuration of $\mathcal {S}$ almost surely reaches some configuration of some successor $\mathcal {S}'$ of $\mathcal {S}$, and, since $\mathcal {S}'$ is inductive, get trapped in $\mathcal {S}'$. A stage graph labels the node $\mathcal {S}$ with a witness of this property in the form of a *Presburger certificate*, a sort of ranking function expressible in Presburger arithmetic. The completeness of the technique, i.e., the fact that for every property of the replicated system that holds there exists a stage graph proving it, follows from deep results of the theory of vector addition systems (VASs) [52–54].

Unfortunately, the theory of VASs also shows that, while the verification problems we consider are decidable, they have non-elementary computational complexity [33]. As a consequence, verification techniques that systematically explore the space of possible stage graphs for a given property are bound to be very inefficient. For this reason, we design an incomplete but efficient algorithm for the computation of stage graphs. Inspired by theoretical results, the algorithm combines a solver for linear constraints with some elements of the theory of well-structured systems [2, 39]. We report on the performance of this algorithm for a large number of case studies. In particular, the algorithm automatically verifies many standard population protocols described in the literature [5, 8, 20, 22, 23, 28, 31], as well as liveness properties of distributed algorithms for leader election and mutual exclusion [3, 40, 42, 44, 50, 59, 61, 64].

*Related Work.* The parameterized verification of replicated systems was first studied in [41], where they were modeled as counter systems. This allows one to apply many efficient techniques [11, 24, 37, 47]. Most of these works are inherently designed for safety properties, and some can also handle fair termination [38], but none of them handles stochastic scheduling. To the best of our knowledge, the only works studying parameterized verification of liveness properties under our notion of stochastic scheduling are those on verification of population protocols. For *fixed* populations, protocols can be verified with standard probabilistic model checking [13, 65], and early works follow this approach [28, 31, 60, 63]. Subsequently, an algorithm and a tool for the *parameterized* verification of population protocols were described in [21, 22], and a first version of stage graphs was introduced in [23] for analyzing the expected termination time of population protocols. In this paper we overhaul the framework of [23] for liveness verification, drawing inspiration from the safety verification technology of [21, 22]. Compared to [21, 22], our approach is not limited to a specific subclass of protocols, and captures models beyond population protocols. Furthermore, our new techniques for computing Presburger certificates subsume the procedure of [22]. In comparison to [23], we provide the first completeness and complexity results for stage graphs. Further, our stage graphs can prove correctness of population protocols and even more general liveness properties, while those of [23] can only prove termination. We also introduce novel techniques for computing stage graphs, which compared to [23] can greatly reduce their size and allows us to prove more examples correct.

There is also a large body of work on parameterized verification via cutoff techniques: one shows that a specification holds for any number of agents iff it holds for any number of agents below some threshold called the cutoff (see [6, 26, 30, 34, 46], and [16] for a comprehensive survey). Cut-off techniques can be applied to systems with an array or ring communication structure, but they require the existence and effectiveness of a cutoff, which is not the case in our setting. Further parameterized verification techniques are regular model checking [1, 25] and automata learning [7]. The classes of communication structures they can handle are orthogonal to ours: arrays and rings for regular model checking and automata learning, and cliques in our work. Regular model checking and learning have recently been employed to verify safety properties [29], liveness properties under arbitrary schedulers [55] and termination under finitary fairness [51]. The classes of schedulers considered in [51, 55] are incomparable to ours: arbitrary schedulers in [55], and finitary-fair schedulers in [51]. Further, these works are based on symbolic state-space exploration, while our techniques are based on automatic construction of invariants and ranking functions [16].

## Preliminaries

Let $\mathbb {N}$ denote $\{0, 1, \ldots \}$ and let *E* be a finite set. A *unordered vector* over *E* is a mapping $V :E \rightarrow \mathbb {Z}$. In particular, a *multiset* over *E* is an unordered vector $M :E \rightarrow \mathbb {N}$ where *M*(*e*) denotes the number of occurrences of *e* in *M*. The sets of all unordered vectors and multisets over *E* are respectively denoted $\mathbb {Z}^E$ and $\mathbb {N}^E$. Vector addition, subtraction and comparison are defined componentwise. The *size* of a multiset *M* is denoted $|M| = \sum _{e \in E} M(e)$. We let $E^{\langle k \rangle }$ denote the set of all multisets over *E* of size *k*. We sometimes describe multisets using a set-like notation, e.g.  or equivalently  is such that $M(f) = 1$, $M(g) = 2$ and $M(e) = 0$ for all $e \not \in \{f, g\}$.

*Presburger Arithmetic.* Let *X* be a set of variables. The set of formulas of *Presburger arithmetic* over *X* is the result of closing atomic formulas, as defined in the next sentence, under Boolean operations and first-order existential quantification. Atomic formulas are of the form $\sum _{i=1}^k a_i x_i \sim b$, where $a_i$ and *b* are integers, $x_i$ are variables and $\sim$ is either < or $\equiv _m$, the latter denoting the congruence modulo *m* for any $m \ge 2$. Formulas over *X* are interpreted on $\mathbb {N}^X$. Given a formula $\phi$ of Presburger arithmetic, we let $\llbracket \phi \rrbracket$ denote the set of all multisets satisfying $\phi$. A set $E \subseteq \mathbb {N}^X$ is a *Presburger set* if $E = \llbracket \phi \rrbracket$ for some formula $\phi$.

### Replicated Systems

A *replicated system* over *Q* of arity *n* is a tuple $\mathcal {P}= (Q,T)$, where $T \subseteq \bigcup _{k=0}^n Q^{\langle k \rangle } \times Q^{\langle k \rangle }$ is a *transition relation* containing the set of *silent* transitions $\bigcup _{k=0}^n \{ (\textit{\textbf{x}}, \textit{\textbf{x}}) \mid \textit{\textbf{x}} \in Q^{\langle k \rangle }) \}$^2^. A *configuration* is a multiset *C* of states, which we interpret as a global state with *C*(*q*) agents in each state $q \in Q$.

For every $t = (\textit{\textbf{x}}, \textit{\textbf{y}}) \in T$ with  and  , we write $X_1 X_2 \cdots X_k \mapsto Y_1 Y_2 \cdots Y_k$ and let ${}^\bullet {t} {\mathop {=}\limits ^{\scriptscriptstyle \text {def}}}\textit{\textbf{x}}$, ${t}^\bullet {\mathop {=}\limits ^{\scriptscriptstyle \text {def}}}\textit{\textbf{y}}$ and $\varDelta (t) {\mathop {=}\limits ^{\scriptscriptstyle \text {def}}}{t}^\bullet - {}^\bullet {t}$. A transition *t* is *enabled* at a configuration *C* if $C \ge {}^\bullet {t}$ and, if so, can *occur*, leading to the configuration $C' = C + \varDelta (t)$. If *t* is not enabled at *C*, then we say that it is *disabled*. We use the following reachability notation: 
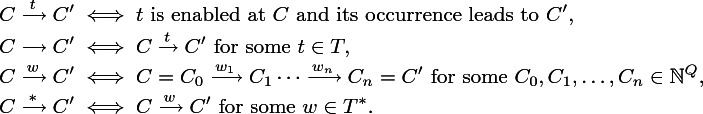
Observe that, by definition of transitions, $C \xrightarrow {} C'$ implies $|C| = |C'|$, and likewise for $C \xrightarrow {*} C'$. Intuitively, transitions cannot create or destroy agents.

A *run* is an infinite sequence $C_0 t_1 C_1 t_2 C_2 \cdots$ such that $C_i \xrightarrow {t_{i+1}} C_{i+1}$ for every $i \ge 0$. Given $L \subseteq T^*$ and a set of configurations $\mathcal {C}$, we let

$$\begin{aligned} post _L(\mathcal {C})&{\mathop {=}\limits ^{\scriptscriptstyle \text {def}}}\{C': C \in \mathcal {C}, w \in L, C \xrightarrow {w} C'\},&post ^*(\mathcal {C})&{\mathop {=}\limits ^{\scriptscriptstyle \text {def}}} post _{T^*}(\mathcal {C}), \\ pre _L(\mathcal {C})&{\mathop {=}\limits ^{\scriptscriptstyle \text {def}}}\{C : C' \in \mathcal {C}, w \in L, C \xrightarrow {w} C'\},&pre ^*(\mathcal {C})&{\mathop {=}\limits ^{\scriptscriptstyle \text {def}}} pre _{T^*}(\mathcal {C}). \end{aligned}$$

*Stochastic Scheduling.* We assume that, given a configuration *C*, a probabilistic scheduler picks one of the transitions enabled at *C*. We only make the following two assumptions about the random experiment determining the transition: first, the probability of a transition depends only on *C*, and, second, every transition enabled at *C* has a nonzero probability of occurring. Since $C \xrightarrow {*} C'$ implies $|C| = |C'|$, the number of configurations reachable from any configuration *C* is finite. Thus, for every configuration *C*, the semantics of $\mathcal {P}$ from *C* is a finite-state Markov chain rooted at *C*.

#### Example 1

Consider the replicated system $\mathcal {P}= (Q,T)$ of arity 2 with states $Q = \{ \text {A}_\text {Y}, \text {A}_\text {N}, \text {P}_\text {Y}, \text {P}_\text {N}\}$ and transitions $T = \{ t_1, t_2, t_3, t_4 \}$, where 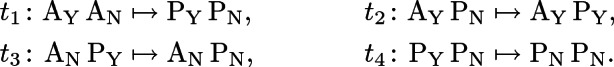
Intuitively, at every moment in time, agents are either *Active* or *Passive*, and have output *Yes* or *No*, which corresponds to the four states of *Q*. This system is designed to satisfy the following property: for every configuration *C* in which all agents are initially active, i.e., *C* satisfies $C(\text {P}_\text {Y})=C(\text {P}_\text {N})=0$, if $C(\text {A}_\text {Y}) > C(\text {A}_\text {N})$, then eventually all agents stay forever in the “yes” states $\{\text {A}_\text {Y}, \text {P}_\text {Y}\}$, and otherwise all agents eventually stay forever in the “no” states $\{\text {A}_\text {N}, \text {P}_\text {N}\}$.    $\triangleleft$

### Qualitative Model Checking

Let us fix a replicated system $\mathcal {P}= (Q,T)$. Formulas of *linear temporal logic (LTL)* on $\mathcal {P}$ are defined by the following grammar:

$$ \varphi \,::= \phi \mid \lnot \varphi \mid \varphi \vee \varphi \mid \varphi \wedge \varphi \mid {\mathbf {X}}\varphi \mid \varphi \mathbin {\mathbf {U}}\varphi $$

where $\phi$ is a Presburger formula over *Q*. We look at $\phi$ as an atomic proposition over the set $\mathbb {N}^Q$ of configurations. Formulas of LTL are interpreted over runs of $\mathcal {P}$ in the standard way. We abbreviate $\Diamond \varphi \equiv \textit{true}\mathbin {\mathbf {U}}\varphi$ and $\Box \varphi \equiv \lnot \Diamond \lnot \varphi$.

Let us now introduce the probabilistic interpretation of LTL. A configuration *C* of $\mathcal {P}$ satisfies an LTL formula $\varphi$ *with probability p* if $\Pr [C, \varphi ] = p$, where $\Pr [C, \varphi ]$ denotes the probability of the set of runs of $\mathcal {P}$ starting at *C* that satisfy $\varphi$ in the finite-state Markov chain rooted at *C*. The measurability of this set of runs for every *C* and $\varphi$ follows from well-known results [65]. The *qualitative model checking problem* consists of, given an LTL formula $\varphi$ and a set of configurations ${\mathcal {I}}$, deciding whether $\Pr [C , \varphi ] = 1$ for every $C \in {\mathcal {I}}$. We will often work with the complement problem, i.e., deciding whether $\Pr [C, \lnot \varphi ] > 0$ for some $C\in {\mathcal {I}}$.

In contrast to the action-based qualitative model checking problem of [35], our version of the problem is undecidable due to adding atomic propositions over configurations (see the full version of the paper [19] for a proof):

#### Theorem 1

The qualitative model checking problem is not semi-decidable.

It is known that qualitative model checking problems of finite-state probabilistic systems reduces to model checking of non-probabilistic systems under an adequate notion of fairness.

#### Definition 1

A run of a replicated system $\mathcal {P}$ is *fair* if for every possible step $C \xrightarrow {t} C'$ of $\mathcal {P}$ the following holds: if the run contains infinitely many occurrences of *C*, then it also contains infinitely many occurrences of $C \, t \, C'$.

So, intuitively, if a run can execute a step infinitely often, it eventually will. It is readily seen that a fair run of a finite-state transition system eventually gets “trapped” in one of its bottom strongly connected components, and visits each of its states infinitely often. Hence, fair runs of a finite-state Markov chain have probability one. The following proposition was proved in [35] for a model slightly less general than replicated systems; the proof can be generalized without effort:

#### Proposition 1

**(** [35, Prop. 7]**).** Let $\mathcal {P}$ be a replicated system, let *C* be a configuration of $\mathcal {P}$, and let $\varphi$ be an LTL formula. It is the case that $\Pr [C, \varphi ] = 1$ iff every fair run of $\mathcal {P}$ starting at *C* satisfies $\varphi$.

We implicitly use this proposition from now on. In particular, we define:

#### Definition 2

A configuration *C* *satisfies* $\varphi$ *with probability 1*, or just *satisfies* $\varphi$, if every fair run starting at *C* satisfies $\varphi$, denoted by $C \models \varphi$. We let $\llbracket \varphi \rrbracket$ denote the set of configurations satisfying $\varphi$. A set $\mathcal {C}$ of configurations *satisfies* $\varphi$ if $\mathcal {C}\subseteq \llbracket \varphi \rrbracket$, i.e., if $C \models \varphi$ for every $C \in \mathcal {C}$.

*Liveness Specifications for Replicated Systems.* We focus on a specific class of temporal properties for which the qualitative model checking problem is decidable and which is large enough to formalize many important specifications. Using well-known automata-theoretic technology, this class can also be used to verify all properties describable in action-based LTL, see e.g. [35].

A *stable termination property* is given by a pair $\varPi = (\varphi _{\mathrm {pre}}, \varPhi _{ post })$, where $\varPhi _{ post }= \{\varphi _{\mathrm {post}}^1, \ldots , \varphi _{\mathrm {post}}^k\}$ and $\varphi _{\mathrm {pre}}, \varphi _{\mathrm {post}}^1, \ldots , \varphi _{\mathrm {post}}^k$ are Presburger formulas over *Q* describing sets of configurations. Whenever $k = 1$, we sometimes simply write $\varPi = (\varphi _{\mathrm {pre}}, \varphi _{\mathrm {post}})$. The pair $\varPi$ induces the LTL property

$$ \varphi _\varPi {\mathop {=}\limits ^{\scriptscriptstyle \text {def}}}\Diamond \bigvee _{i=1}^k \Box \varphi _{\mathrm {post}}^i. $$

Abusing language, we say that a replicated system $\mathcal {P}$ *satisfies* $\varPi$ if $\llbracket \varphi _{\mathrm {pre}}\rrbracket \subseteq \llbracket \varphi _\varPi \rrbracket$, that is, if every configuration *C* satisfying $\varphi _{\mathrm {pre}}$ satisfies $\varphi _\varPi$ with probability 1.

The *stable termination problem* is the qualitative model checking problem for ${\mathcal {I}}= \llbracket \varphi _{\mathrm {pre}}\rrbracket$ and $\varphi = \varphi _\varPi$ given by a stable termination property $\varPi = (\varphi _{\mathrm {pre}}, \varPhi _{ post })$.

#### Example 2

Let us reconsider the system from Example 1. We can formally specify that all agents will eventually agree on the majority output *Yes* or *No*. Let $\varPi ^{\text {Y}} = (\varphi _{\mathrm {pre}}^{\text {Y}}, \varphi _{\mathrm {post}}^{\text {Y}})$ and $\varPi ^{\text {N}} = (\varphi _{\mathrm {pre}}^{\text {N}}, \varphi _{\mathrm {post}}^{\text {N}})$ be defined by:

$$\begin{aligned} \varphi _{\mathrm {pre}}^{\text {Y}}&= (\text {A}_\text {Y}> \text {A}_\text {N}\wedge \text {P}_\text {Y}+ \text {P}_\text {N}= 0),&\varphi _{\mathrm {post}}^{\text {Y}}&= (\text {A}_\text {N}+ \text {P}_\text {N}= 0), \\ \varphi _{\mathrm {pre}}^{\text {N}}&= (\text {A}_\text {Y}\le \text {A}_\text {N}\wedge \text {P}_\text {Y}+ \text {P}_\text {N}= 0),&\varphi _{\mathrm {post}}^{\text {N}}&= (\text {A}_\text {Y}+ \text {P}_\text {Y}= 0). \end{aligned}$$

The system satisfies the property specified in Example 1 iff it satisfies $\varPi ^{\text {Y}}$ and $\varPi ^{\text {N}}$. As an alternative (weaker) property, we could specify that the system always stabilizes to either output by $\varPi = (\varphi _{\mathrm {pre}}^{\text {Y}} \vee \varphi _{\mathrm {pre}}^{\text {N}}, \{ \varphi _{\mathrm {post}}^{\text {Y}}, \varphi _{\mathrm {post}}^{\text {N}} \})$.    $\triangleleft$

## Stage Graphs

In the rest of the paper, we fix a replicated system $\mathcal {P}= (Q,T)$ and a stable termination property $\varPi = (\varphi _{\mathrm {pre}}, \varPhi _{ post })$, where $\varPhi _{ post }= \{\varphi _{\mathrm {post}}^1, \ldots , \varphi _{\mathrm {post}}^k\}$, and address the problem of checking whether $\mathcal {P}$ satisfies $\varPi$. We start with some basic definitions on sets of configurations.

### Definition 3

**(inductive sets, leads to, certificates)**

- A set of configurations $\mathcal {C}$ is *inductive* if $C \in \mathcal {C}$ and $C \rightarrow C'$ implies $C' \in \mathcal {C}$.
- Let $\mathcal {C}, \mathcal {C}'$ be sets of configurations. We say that $\mathcal {C}$ *leads to* $\mathcal {C}'$, denoted $\mathcal {C}\leadsto \mathcal {C}'$, if for all $C \in \mathcal {C}$, every fair run from *C* eventually visits a configuration of $\mathcal {C}'$.
- A *certificate* for $\mathcal {C}\leadsto \mathcal {C}'$ is a function $f :\mathcal {C}\rightarrow \mathbb {N}$ satisfying that for every $C \in \mathcal {C}\setminus \mathcal {C}'$, there exists an execution $C \xrightarrow {*} C'$ such that $f(C) > f(C')$.

Note that certificates only require the existence of some executions decreasing *f*, not for all of them to to decrease it. Despite this, we have:

### Proposition 2

For all inductive sets $\mathcal {C}, \mathcal {C}'$ of configurations, it is the case that: $\mathcal {C}$ leads to $\mathcal {C}'$ iff there exists a certificate for $\mathcal {C}\leadsto \mathcal {C}'$.

The proof, which can be found in the full version [19], depends on two properties of replicated systems with stochastic scheduling. First, every configuration has only finitely many descendants. Second, for every fair run and for every finite execution $C \xrightarrow {w} C'$, if *C* appears infinitely often in the run, then the run contains infinitely many occurrences of $C \xrightarrow {w} C'$. We can now introduce stage graphs:

### Definition 4

**(stage graph).** A *stage graph* of $\mathcal {P}$ for the property $\varPi$ is a directed acyclic graph whose nodes, called *stages*, are sets of configurations satisfying the following conditions:

1. every stage is an inductive set;
2. every configuration of $\llbracket \varphi _{\mathrm {pre}}\rrbracket$ belongs to some stage;
3. if $\mathcal {C}$ is a non-terminal stage with successors $\mathcal {C}_1, \ldots , \mathcal {C}_n$, then there exists a certificate for $\mathcal {C}\leadsto (\mathcal {C}_1 \cup \cdots \cup \mathcal {C}_n)$;
4. if $\mathcal {C}$ is a terminal stage, then $\mathcal {C}\models \varphi _{\mathrm {post}}^i$ for some *i*.

The existence of a stage graph implies that $\mathcal {P}$ satisfies $\varPi$. Indeed, by conditions 2–3 and repeated application of Proposition 2, every run starting at a configuration of $\llbracket \varphi _{\mathrm {pre}}\rrbracket$ eventually reaches a terminal stage, say $\mathcal {C}$, and, by condition 1, stays in $\mathcal {C}$ forever. Since, by condition 4, all configurations of $\mathcal {C}$ satisfy some $\varphi _{\mathrm {post}}^i$, after its first visit to $\mathcal {C}$ every configuration satisfies $\varphi _{\mathrm {post}}^i$.

**Fig. 1.**
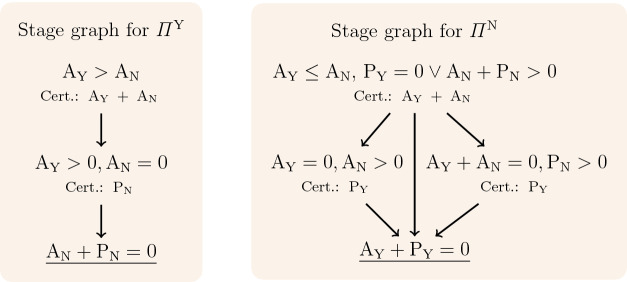
Stage graphs for the system of Example 1.

### Example 3

Figure 1 depicts stage graphs for the system of Example 1 and the properties defined in Example 2. The reader can easily show that every stage $\mathcal {C}$ is inductive by checking that for every $C \in \mathcal {C}$ and every transition $t \in \{t_1, \ldots , t_4\}$ enabled at *C*, the step $C \xrightarrow {t_i} C'$ satisfies $C' \in \mathcal {C}$. For example, if a configuration satisfies $\text {A}_\text {Y}> \text {A}_\text {N}$, so does any successor configuration.    $\triangleleft$

The following proposition shows that stage graphs are a sound and complete technique for proving stable termination properties.

### Proposition 3

System $\mathcal {P}$ satisfies $\varPi$ iff it has a stage graph for $\varPi$.

Proposition 3 does not tell us anything about the decidability of the stable termination problem. To prove that the problem is decidable, we introduce Presburger stage graphs. Intuitively these are stage graphs whose stages and certificates can be expressed by formulas of Presburger arithmetic.

### Definition 5

**(Presburger stage graphs)**

- A stage $\mathcal {C}$ is *Presburger* if $\mathcal {C}= \llbracket \phi \rrbracket$ for some Presburger formula $\phi$.
- A *bounded certificate* for $\mathcal {C}\leadsto \mathcal {C}'$ is a pair (*f*, *k*), where $f :\mathcal {C}\rightarrow \mathbb {N}$ and $k \in \mathbb {N}$, satisfying that for every $C \in \mathcal {C}\setminus \mathcal {C}'$, there exists an execution $C \xrightarrow {w} C'$ such that $f(C) > f(C')$ and $|w| \le k$.
- A *Presburger certificate* is a bounded certificate (*f*, *k*) satisfying $f(C)= n \iff \varphi (C,n)$ for some Presburger formula $\varphi (\textit{\textbf{x}}, y)$.
- A *Presburger stage graph* is a stage graph whose stages and certificates are all Presburger.

Using a powerful result from [36], we show that: (1) $\mathcal {P}$ satisfies $\varPi$ iff it has a Presburger stage graph for $\varPi$ (Theorem 2); (2) there exists a denumerable set of candidates for a Presburger stage graph for $\varPi$; and (3) there is an algorithm that decides whether a given candidate is a Presburger stage graph for $\varPi$ (Theorem 3). Together, (1–3) show that the stable termination problem is semi-decidable. To obtain decidability, we observe that the complement of the stable termination problem is also semi-decidable. Indeed, it suffices to enumerate all initial configurations $C \models \varphi _{\mathrm {pre}}$, build for each such *C* the (finite) graph $G_C$ of configurations reachable from *C*, and check if some bottom strongly connected component $\mathcal {B}$ of $G_C$ satisfies $\mathcal {B}\not \models \varphi _{\mathrm {post}}^i$ for all *i*. This is the case iff some fair run starting at *C* visits and stays in $\mathcal {B}$, which in turn is the case iff $\mathcal {P}$ violates $\varPi$.

### Theorem 2

System $\mathcal {P}$ satisfies $\varPi$ iff it has a Presburger stage graph for $\varPi$.

We observe that testing whether a given graph is a Presburger stage graph reduces to Presburger arithmetic satisfiability, which is decidable [62] and whose complexity lies between 2-NEXP and 2-EXPSPACE [15]:

### Theorem 3

The problem of deciding whether an acyclic graph of Presburger sets and Presburger certificates is a Presburger stage graph, for a given stable termination property, is reducible in polynomial time to the satisfiability problem for Presburger arithmetic.

## Algorithmic Construction of Stage Graphs

At the current state of our knowledge, the decision procedure derived from Theorem 3 has little practical relevance. From a theoretical point of view, the TOWER-hardness result of [33] implies that the stage graph may have non-elementary size in the system size. In practice, systems have relatively small stage graphs, but, even so, the enumeration of all candidates immediately leads to a prohibitive combinatorial explosion.

For this reason, we present a procedure to automatically *construct* (not guess) a Presburger stage graph *G* for a given replicated system $\mathcal {P}$ and a stable termination property $\varPi = (\varphi _{\mathrm {pre}}, \varPhi _{ post })$. The procedure may *fail*, but, as shown in the experimental section, it succeeds for many systems from the literature.

The procedure is designed to be implemented on top of a solver for the existential fragment of Presburger arithmetic. While every formula of Presburger arithmetic has an equivalent formula within the existential fragment [32, 62], quantifier-elimination may lead to a doubly-exponential blow-up in the size of the formula. Thus, it is important to emphasize that our procedure *never requires to eliminate quantifiers*: If the pre- and postconditions of $\varPi$ are supplied as quantifier-free formulas, then all constraints of the procedure remain in the existential fragment.

We give a high-level view of the procedure (see Algorithm 1), which uses several functions, described in detail in the rest of the paper. The procedure maintains a workset $WS$ of Presburger stages, represented by existential Presburger formulas. Initially, the only stage is an inductive Presburger overapproximation $\textit{PotReach}(\llbracket \varphi _{\mathrm {pre}}\rrbracket )$ of the configurations reachable from $\llbracket \varphi _{\mathrm {pre}}\rrbracket$ ($\textit{PotReach}$ is an abbreviation for “potentially reachable”). Notice that we must necessarily use an overapproximation, since $post ^*(\llbracket \varphi _{\mathrm {pre}}\rrbracket )$ is not always expressible in Presburger arithmetic^3^. We use a refinement of the overapproximation introduced in [22, 37], equivalent to the overapproximation of [24].
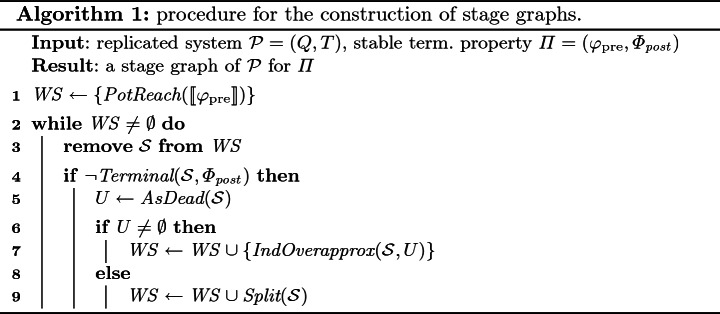


In its main loop (lines 2–9), Algorithm 1 picks a Presburger stage $\mathcal {S}$ from the workset, and processes it. First, it calls $\text {Terminal}(\mathcal {S},\varPhi _{ post })$ to check if $\mathcal {S}$ is terminal, i.e., whether $\mathcal {S}\models \varphi _{\mathrm {post}}^i$ for some $\varphi _{\mathrm {post}}^i \in \varPhi _{ post }$. This reduces to checking the unsatisfiability of the existential Presburger formula $\phi \wedge \lnot \varphi _{\mathrm {post}}^i$, where $\phi$ is the formula characterizing $\mathcal {S}$. If $\mathcal {S}$ is not terminal, then the procedure attempts to construct successor stages in lines 5–9, with the help of three further functions: $\textit{AsDead}$, $\textit{IndOverapprox}$, and $\textit{Split}$. In the rest of this section, we present the intuition behind lines 5–9, and the specification of the three functions. Sections 5, 6 and 7 present the implementations we use for these functions.

Lines 5–9 are inspired by the behavior of most replicated systems designed by humans, and are based on the notion of *dead* transitions, which can never occur again (to be formally defined below). Replicated systems are usually designed to run in *phases*. Initially, all transitions are alive, and the end of a phase is marked by the “death” of one or more transitions, i.e., by reaching a configuration at which these transitions are dead. The system keeps “killing transitions” until no transition that is still alive can lead to a configuration violating the postcondition. The procedure mimics this pattern. It constructs stage graphs in which if $\mathcal {S}'$ is a successor of $\mathcal {S}$, then the set of transitions dead at $\mathcal {S}'$ is a *proper superset* of the transitions dead at $\mathcal {S}$. For this, $\textit{AsDead}(\mathcal {S})$ computes a set of transitions that are alive at some configuration of $\mathcal {S}$, but which will become dead in every fair run starting at $\mathcal {S}$ (line 5). Formally, $\textit{AsDead}(\mathcal {S})$ returns a set $U \subseteq \overline{\textit{Dead}(\mathcal {S})}$ such that $\mathcal {S}\models \Diamond \text {dead}(U)$, defined as follows.

### Definition 6

A transition of a replicated system $\mathcal {P}$ is *dead* at a configuration *C* if it is disabled at every configuration reachable from *C* (including *C* itself). A transition is *dead* at a stage $\mathcal {S}$ if it is dead at every configuration of $\mathcal {S}$. Given a stage $\mathcal {S}$ and a set *U* of transitions, we use the following notations:

- $\textit{Dead}(\mathcal {S})$: the set of transitions dead at $\mathcal {S}$;
- $\llbracket \text {dis}(U)\rrbracket$: the set of configurations at which all transitions of *U* are disabled;
- $\llbracket \text {dead}(U)\rrbracket$: the set of configurations at which all transitions of *U* are dead.

Observe that we can compute $\textit{Dead}(\mathcal {S})$ by checking unsatisfiability of a sequence of existential Presburger formulas: as $\mathcal {S}$ is inductive, we have $\textit{Dead}(\mathcal {S}) = \{ t \mid \mathcal {S}\models \text {dis}(t) \}$, and $\mathcal {S}\models \text {dis}(t)$ holds iff the existential Presburger formula $\exists C :\phi (C) \wedge C \ge {}^\bullet {t}$ is unsatisfiable, where $\phi$ is the formula characterizing $\mathcal {S}$.

The following proposition, whose proof appears in the full version [19], shows that determining whether a given transition will eventually become dead, while decidable, is PSPACE-hard. Therefore, Sect. 7 describes two implementations of this function, and a way to combine them, which exhibit a good trade-off between precision and computation time.

### Proposition 4

Given a replicated system $\mathcal {P}$, a stage $\mathcal {S}$ represented by an existential Presburger formula $\phi$ and a set of transitions *U*, determining whether $\mathcal {S}\models \Diamond \text {dead}(U)$ holds is decidable and PSPACE-hard.

If the set *U* returned by $\textit{AsDead}(\mathcal {S})$ is nonempty, then we know that every fair run starting at a configuration of $\mathcal {S}$ will eventually reach a configuration of $\mathcal {S}\cap \llbracket \text {dead}(U)\rrbracket$. So, this set, or any inductive overapproximation of it, can be a legal successor of $\mathcal {S}$ in the stage graph. Function $\textit{IndOverapprox}(\mathcal {S},U)$ returns such an inductive overapproximation (line 7). To be precise, we show in Sect. 5 that $\llbracket \text {dead}(U)\rrbracket$ is a Presburger set that can be computed exactly, albeit in doubly-exponential time in the worst case. The section also shows how to compute overapproximations more efficiently. If the set *U* returned by $\textit{AsDead}(\mathcal {S})$ is empty, then we cannot yet construct any successor of $\mathcal {S}$. Indeed, recall that we want to construct stage graphs in which if $\mathcal {S}'$ is a successor of $\mathcal {S}$, then $\textit{Dead}(\mathcal {S}')$ is a *proper superset* of $\textit{Dead}(\mathcal {S})$. In this case, we proceed differently and try to split $\mathcal {S}$:

### Definition 7

A *split* of some stage $\mathcal {S}$ is a set $\{\mathcal {S}_1, \ldots , \mathcal {S}_k\}$ of (not necessarily disjoint) stages such that the following holds:

- $\textit{Dead}(\mathcal {S}_i) \supset \textit{Dead}(\mathcal {S})$ for every $1 \le i \le k$, and
- $$\mathcal {S}= \bigcup _{i=1}^k \mathcal {S}_i$$.

If there exists a split $\{\mathcal {S}_1, \ldots , \mathcal {S}_k\}$ of $\mathcal {S}$, then we can let $\mathcal {S}_1, \ldots , \mathcal {S}_k$ be the successors of $\mathcal {S}$ in the stage graph. Observe that a stage may indeed have a split. We have $\textit{Dead}(\mathcal {C}_1 \cup \mathcal {C}_2) = \textit{Dead}(\mathcal {C}_1) \cap \textit{Dead}(\mathcal {C}_2)$, and hence $\textit{Dead}(\mathcal {C}_1 \cup \mathcal {C}_2)$ may be a proper subset of both $\textit{Dead}(\mathcal {C}_1)$ and $\textit{Dead}(\mathcal {C}_2)$:

### Example 4

Consider the system with states $\{q_1, q_2\}$ and transitions $t_i :q_i \mapsto q_i$ for $i \in \{1, 2\}$. Let $\mathcal {S}= \{ C \mid C(q_1) = 0 \vee C(q_2) = 0 \}$, i.e., $\mathcal {S}$ is the (inductive) stage of configurations disabling either $t_1$ or $t_2$. The set $\{ \mathcal {S}_1, \mathcal {S}_2 \}$, where $\mathcal {S}_i = \{ C \in \mathcal {S}\mid C(q_i) = 0 \}$, is a split of $\mathcal {S}$ satisfying $\textit{Dead}(\mathcal {S}_i) = \{t_i\} \supset \emptyset = \textit{Dead}(\mathcal {S})$. $\triangleleft$

The canonical split of $\mathcal {S}$, if it exists, is the set $\{ \mathcal {S}\cap \llbracket \text {dead}(t)\rrbracket \mid t \notin \textit{Dead}(\mathcal {S}) \}$. As mentioned above, Sect. 5 shows that $\llbracket \text {dead}(U)\rrbracket$ can be computed exactly for every *U*, but the computation can be expensive. Hence, the canonical split can be computed exactly at potentially high cost. Our implementation uses an underapproximation of $\llbracket \text {dead}(t)\rrbracket$, described in Sect. 6.

## Computing and Approximating $\llbracket \text {dead}(U)\rrbracket$

We show that, given a set *U* of transitions,

- we can effectively compute an existential Presburger formula describing the set $\llbracket \text {dead}(U)\rrbracket$, with high computational cost in the worst case, and
- we can effectively compute constraints that overapproximate or underapproximate $\llbracket \text {dead}(U)\rrbracket$, at a reduced computational cost.

**Downward and Upward Closed Sets.** We enrich $\mathbb {N}$ with the limit element $\omega$ in the usual way. In particular, $n < \omega$ holds for every $n \in \mathbb {N}$. An $\omega$*-configuration* is a mapping $C^\omega :Q \rightarrow \mathbb {N}\cup \{\omega \}$. The *upward closure* and *downward closure* of a set $\mathcal {C}^\omega$ of $\omega$-configurations are the sets of configurations  and , respectively defined as:A set $\mathcal {C}$ of configurations is *upward closed* if , and *downward closed* if . These facts are well-known from the theory of well-quasi orderings:

### Lemma 1

For every set $\mathcal {C}$ of configurations, the following holds:

1. $\mathcal {C}$ is upward closed iff $\overline{\mathcal {C}}$ is downward closed (and vice versa);
2. if $\mathcal {C}$ is upward closed, then there is a unique minimal finite set of configurations $\text {inf}(\mathcal {C})$, called its *basis*, such that ;
3. if $\mathcal {C}$ is downward closed, then there is a unique minimal finite set of $\omega$-configurations $\text {sup}(\mathcal {C})$, called its *decomposition*, such that .

**Computing** $\varvec{\llbracket \text {dead}(U)\rrbracket }$ **Exactly.** It follows immediately from Definition 6 that both $\llbracket \text {dis}(U)\rrbracket$ and $\llbracket \text {dead}(U)\rrbracket$ are downward closed. Indeed, if all transitions of *U* are disabled at *C*, and $C' \le C$, then they are also disabled at $C'$, and clearly the same holds for transitions dead at *C*. Furthermore:

### Proposition 5

For every set *U* of transitions, the (downward) decomposition of both $\text {sup}(\llbracket \text {dis}(U)\rrbracket )$ and $\text {sup}(\llbracket \text {dead}(U)\rrbracket )$ is effectively computable.

### Proof

For every $t \in U$ and $q \in {}^\bullet {t}$, let $C_{t, q}^\omega$ be the $\omega$-configuration such that $C_{t, q}^\omega (q) = {}^\bullet {t}(q) - 1$ and $C_{t, q}^\omega (p) = \omega$ for every $p \in Q \setminus \{q\}$. In other words, $C_{t, q}^\omega$ is the $\omega$-configuration made only of $\omega$’s except for state *q* which falls short from ${}^\bullet {t}(q)$ by one. This $\omega$-configurations captures all configurations disabled in *t* due to an insufficient amount of agents in state *q*. We have:

$$\text {sup}(\llbracket \text {dis}(U)\rrbracket ) = \{C_{t, q}^\omega : t \in U, q \in {}^\bullet {t}\}.$$

The latter can be made minimal by removing superfluous $\omega$-configurations.

For the case of $\text {sup}(\llbracket \text {dead}(U)\rrbracket )$, we invoke [45, Prop. 2] which gives a proof for the more general setting of (possibly unbounded) Petri nets. Their procedure is based on the well-known backwards reachability algorithm (see, e.g., [2, 39]).    $\square$

Since $\text {sup}(\llbracket \text {dead}(U)\rrbracket )$ is finite, its computation allows to describe $\llbracket \text {dead}(U)\rrbracket$ by the following linear constraint^4^:

$$\bigvee _{C^\omega \in \text {sup}(\llbracket \text {dead}(U)\rrbracket )} \bigwedge _{q \in Q} \left[ C(q) \le C^\omega (q)\right] .$$

However, the cardinality of $\text {sup}(\llbracket \text {dead}(U)\rrbracket )$ can be exponential [45, Remark for Prop. 2] in the system size. For this reason, we are interested in constructing both under- and over-approximations.

**Overapproximations of** $\varvec{\llbracket \text {dead}(U)\rrbracket }$**.** For every $i \in \mathbb {N}$, define $\llbracket \text {dead}(U)\rrbracket ^i$ as:

$$ \llbracket \text {dead}(U)\rrbracket ^0 {\mathop {=}\limits ^{\scriptscriptstyle \text {def}}}\llbracket \text {dis}(U)\rrbracket \quad \text { and } \quad \llbracket \text {dead}(U)\rrbracket ^{i+1} {\mathop {=}\limits ^{\scriptscriptstyle \text {def}}}\overline{ pre _T(\overline{\llbracket \text {dead}(U)\rrbracket ^{i}})} \cap \llbracket \text {dis}(U)\rrbracket . $$

Loosely speaking, $\llbracket \text {dead}(U)\rrbracket ^{i}$ is the set of configurations *C* such that every configuration reachable in at most *i* steps from *C* disables *U*. We immediately have:

$$ \llbracket \text {dead}(U)\rrbracket = \bigcap _{i=0}^\infty \llbracket \text {dead}(U)\rrbracket ^{i}. $$

Using Proposition 5 and the following proposition, we obtain that $\llbracket \text {dead}(U)\rrbracket ^{i}$ is an effectively computable overapproximation of $\llbracket \text {dead}(U)\rrbracket$.

### Proposition 6

For every Presburger set $\mathcal {C}$ and every set of transitions *U*, the sets $pre _U(\mathcal {C})$ and $post _U(\mathcal {C})$ are effectively Presburger.

Recall that function $\textit{IndOverapprox}(\mathcal {S}, U)$ of Algorithm 1 must return an *inductive* overapproximation of $\llbracket \text {dead}(U)\rrbracket$. Since $\llbracket \text {dead}(U)\rrbracket ^i$ might not be inductive in general, our implementation uses either the inductive overapproximations $\textit{IndOverapprox}^i(\mathcal {S}, U) {\mathop {=}\limits ^{\scriptscriptstyle \text {def}}}\textit{PotReach}(\mathcal {S}\cap \llbracket \text {dead}(U)\rrbracket ^i)$, or the exact value $\textit{IndOverapprox}^\infty (\mathcal {S}, U) {\mathop {=}\limits ^{\scriptscriptstyle \text {def}}}\mathcal {S}\cap \llbracket \text {dead}(U)\rrbracket$. The table of results in the experimental section describes for each benchmark which overapproximation was used.

**Underapproximations of** $\varvec{\llbracket \text {dead}(U)\rrbracket }$**: Death Certificates.** A *death certificate* for *U* in $\mathcal {P}$ is a finite set $\mathcal {C}^\omega$ of $\omega$-configurations such that:

, i.e., every configuration of  disables *U*, and
is inductive, i.e., .

If *U* is dead at a set $\mathcal {C}$ of configurations, then there is always a certificate that proves it, namely $\text {sup}(\llbracket \text {dead}(U)\rrbracket )$. In particular, if $\mathcal {C}^\omega$ is a death certificate for *U* then , that is,  is an underapproximation of $\llbracket \text {dead}(U)\rrbracket$

Using Proposition 6, it is straightforward to express in Presburger arithmetic that a finite set $\mathcal {C}^\omega$ of $\omega$-configurations is a death certificate for *U*:

### Proposition 7

For every $k \ge 1$ there is an existential Presburger formula $DeathCert _k(U, \mathcal {C}^\omega )$ that holds iff $\mathcal {C}^\omega$ is a death certificate of size *k* for *U*.

## Splitting a Stage

Given a stage $\mathcal {S}$, we try to find a set $\mathcal {C}^\omega _1, \ldots , \mathcal {C}^\omega _\ell$ of death certificates for transitions $t_1, \ldots , t_\ell \in T \setminus \textit{Dead}(\mathcal {S})$ such that . This allows us to split $\mathcal {S}$ into $\mathcal {S}_1, \ldots , \mathcal {S}_\ell$, where .

For any fixed size $k \ge 1$ and any fixed $\ell$, we can find death certificates $\mathcal {C}^\omega _1, \ldots , \mathcal {C}^\omega _\ell$ of size at most *k* by solving a Presburger formula. However, the formula does not belong to the existential fragment, because the inclusion check  requires universal quantification. For this reason, we proceed iteratively. For every $i \ge 0$, after having found $\mathcal {C}^\omega _1, \ldots , \mathcal {C}^\omega _i$ we search for a pair $(C_{i+1}, \mathcal {C}^\omega _{i+1})$ such that

(i) $\mathcal {C}^\omega _{i+1}$ is a death certificate for some $t_{i+1} \in T \setminus \textit{Dead}(\mathcal {S})$;
(ii).

An efficient implementation requires to guide the search for $(C_{i+1}, \mathcal {C}^\omega _{i+1})$, because otherwise the search procedure might not even terminate, or might split $\mathcal {S}$ into too many parts, blowing up the size of the stage graph. Our search procedure employs the following heuristic, which works well in practice. We only consider the case $k=1$, and search for a pair $(C_{i+1}, C^\omega _{i+1})$ satisfying (i) and (ii) above, and additionally:

(iii) all components of $C^\omega _{i+1}$ are either $\omega$ or between 0 and $\max _{t \in T,q \in Q} {}^\bullet {t}(q)-1$;
(iv) for every $\omega$-configuration $C^ \omega$, if $(C_{i+1}, C^\omega )$ satisfies (i)–(iii), then $C^\omega _{i+1} \le C^\omega$;
(v) for every pair $(C, C^\omega )$, if $(C, C^\omega )$ satisfies (i)–(iv), then $C^\omega \le C^\omega _{i+1}$.

Condition (iii) guarantees termination. Intuitively, condition (iv) leads to certificates valid for sets $U \subseteq T \setminus \textit{Dead}(\mathcal {S})$ as large as possible. So it allows us to avoid splits that, loosely speaking, do not make as much progress as they could. Condition (v) allows us to avoid splits with many elements because each element of the split has a small intersection with $\mathcal {S}$.

An example illustrating these conditions is given in the full version [19].

## Computing Eventually Dead Transitions

Recall that the function $\textit{AsDead}(\mathcal {S})$ takes an inductive Presburger set $\mathcal {S}$ as input, and returns a (possibly empty) set $U \subseteq \overline{\textit{Dead}(\mathcal {S})}$ of transitions such that $\mathcal {S}\models \Diamond \text {dead}(U)$. This guarantees $\mathcal {S}\leadsto \llbracket \text {dead}(U)\rrbracket$ and, since $\mathcal {S}$ is inductive, also $\mathcal {S}\leadsto \mathcal {S}\cap \llbracket \text {dead}(U)\rrbracket$.

By Proposition 4, deciding if there exists a non-empty set *U* of transitions such that $\mathcal {S}\models \Diamond \text {dead}(U)$ holds is PSPACE-hard, which makes a polynomial reduction to satisfiability of existential Presburger formulas unlikely. So we design incomplete implementations of $\textit{AsDead}(\mathcal {S})$ with lower complexity. Combining these implementations, the lack of completeness essentially vanishes in practice.

The implementations are inspired by Proposition 2, which shows that $\mathcal {S}\leadsto \llbracket \text {dead}(U)\rrbracket$ holds iff there exists a certificate *f* such that:

Cert $$\begin{aligned}\forall C \in \mathcal {S}\setminus \llbracket \text {dead}(U)\rrbracket \ :\exists \, C \xrightarrow {*} C' :f(C) > f(C'). \end{aligned}$$

To find such certificates efficiently, we only search for *linear* functions $f(C) = \sum _{q \in Q} \textit{\textbf{a}}(q) \cdot C(q)$ with coefficients $\textit{\textbf{a}}(q) \in \mathbb {N}$ for each $q \in Q$.

### First Implementation: Linear Ranking Functions

Our first procedure computes the existence of a linear *ranking function*.

#### Definition 8

A function $r :\mathcal {S}\rightarrow \mathbb {N}$ is a ranking function for $\mathcal {S}$ and *U* if for every $C \in \mathcal {S}$ and every step $C \xrightarrow {t} C'$ the following holds:

1. if $t \in U$, then $r(C) > r(C')$; and
2. if $$t \notin U$$, then $$r(C) \ge r(C')$$.

#### Proposition 8

If $r :\mathcal {S}\rightarrow \mathbb {N}$ is a ranking function for $\mathcal {S}$ and *U*, then there exists $k \in \mathbb {N}$ such that (*r*, *k*) is a bounded certificate for $\mathcal {S}\leadsto \llbracket \text {dead}(U)\rrbracket$.

#### Proof

Let *M* be the minimal finite basis of the upward closed set $\overline{\llbracket \text {dead}(U)\rrbracket }$. For every configuration $D \in M$, let $\sigma _D$ be a shortest sequence that enables some transition of $t_D \in U$ from *D*, i.e., such that $D \xrightarrow {\sigma _D} D' \xrightarrow {t_D} D''$ for some $D'$, $D''$. Let $k {\mathop {=}\limits ^{\scriptscriptstyle \text {def}}}\max \{|\sigma _D t_D| : D \in M\}$.

Let $C \in \mathcal {S}\setminus \llbracket \text {dead}(U)\rrbracket$. Since $C \in \overline{\llbracket \text {dead}(U)\rrbracket }$, we have $C \ge D$ for some $D \in M$. By monotonicity, we have $C \xrightarrow {\sigma _D} C' \xrightarrow {t_D} C''$ for some configurations $C'$ and $C''$. By Definition 8, we have $r(C) \ge r(C') > r(C'')$, and so condition (Cert) holds. As $|\sigma _D t_D| \le k$, we have that (*r*, *k*) is a bounded certificate.    $\square$

It follows immediately from Definition 8 that if $r_1$ and $r_2$ are ranking functions for sets $U_1$ and $U_2$ respectively, then *r* defined as $r(C) {\mathop {=}\limits ^{\scriptscriptstyle \text {def}}}r_1(C) + r_2(C)$ is a ranking function for $U_1 \cup U_2$. Therefore, there exists a unique maximal set of transitions *U* such that $\mathcal {S}\leadsto \llbracket \text {dead}(U)\rrbracket$ can be proved by means of a ranking function. Further, *U* can be computed by collecting all transitions $t \in \overline{\textit{Dead}(\mathcal {S})}$ such that there exists a ranking function $r_t$ for $\{t\}$. The existence of a *linear* ranking function $r_t$ can be decided in polynomial time via linear programming, as follows. Recall that for every step $C \xrightarrow {u} C'$, we have $C' = C + \varDelta (u)$. So, by linearity, we have $r_t(C) \ge r_t(C') \iff r_t(C' - C) \le 0 \iff r_t(\varDelta (u)) \le 0$. Thus, the constraints of Definition 8 can be specified as:

$$\begin{aligned} \textit{\textbf{a}} \cdot \varDelta (t) < 0 \quad \wedge \bigwedge _{u \in \overline{\textit{Dead}(\mathcal {S})}} \textit{\textbf{a}} \cdot \varDelta (u) \le 0 , \end{aligned}$$

where $\textit{\textbf{a}} :Q \rightarrow \mathbb {Q}_{\ge 0}$ gives the coefficients of $r_t$, that is, $r_t(C) = \textit{\textbf{a}} \cdot C$, and $\textit{\textbf{a}} \cdot \textit{\textbf{x}} {\mathop {=}\limits ^{\scriptscriptstyle \text {def}}}\sum _{q \in Q} \textit{\textbf{a}}(q) \cdot \textit{\textbf{x}}(q)$ for $\textit{\textbf{x}} \in \mathbb {N}^Q$. Observe that a solution may yield a function whose codomain differs from $\mathbb {N}$. However, this is not an issue since we can scale it with the least common denominator of each $\textit{\textbf{a}}(q)$.

### Second Implementation: Layers

*Transitions layers* were introduced in [22] as a technique to find transitions that will eventually become dead. Intuitively, a set *U* of transitions is a layer if (1) no run can contain only transitions of *U*, and (2) *U* becomes dead once disabled; the first condition guarantees that *U* eventually becomes disabled, and the second that it eventually becomes dead. We formalize layers in terms of *layer functions*.

#### Definition 9

A function $\ell :\mathcal {S}\rightarrow \mathbb {N}$ is a *layer function* for $\mathcal {S}$ and *U* if:

**C1.**: $\ell (C) > \ell (C')$ for every $C \in \mathcal {S}$ and every step $C \xrightarrow {t} C'$ with $t \in U$; and
**C2.**: $$\llbracket \text {dis}(U)\rrbracket = \llbracket \text {dead}(U)\rrbracket $$.

#### Proposition 9

If $\ell :\mathcal {S}\rightarrow \mathbb {N}$ is a layer function for $\mathcal {S}$ and *U*, then $(\ell , 1)$ is a bounded certificate for $\mathcal {S}\leadsto \llbracket \text {dead}(U)\rrbracket$.

#### Proof

Let $C \in \mathcal {S}\setminus \llbracket \text {dead}(U)\rrbracket$. By condition **C2**, we have $C \not \in \llbracket \text {dis}(U)\rrbracket$. So there exists a step $C \xrightarrow {u} C'$ where $u \in U$. By condition **C1**, we have $\ell (C) > \ell (C')$, so condition (Cert) holds and $(\ell , 1)$ is a bounded certificate.

Let $\mathcal {S}$ be a stage. For every set of transitions $U \subseteq \overline{\textit{Dead}(\mathcal {S})}$ we can construct a Presburger formula $\textit{lin-layer}(U, \textit{\textbf{a}})$ that holds iff there there exists a *linear* layer function for *U*, i.e., a layer function of the form $\ell (C) = \textit{\textbf{a}} \cdot C$ for a vector of coefficients $\textit{\textbf{a}} :Q \rightarrow \mathbb {Q}_{\ge 0}$. Condition **C1**, for a linear function $\ell (C)$, is expressed by the existential Presburger formula

$$\begin{aligned} \textit{lin-layer-fun}(U, \textit{\textbf{a}}) {\mathop {=}\limits ^{\scriptscriptstyle \text {def}}}\bigwedge _{u \in U} \textit{\textbf{a}} \cdot \varDelta (u) < 0. \end{aligned}$$

Condition **C2** is expressible in Presburger arithmetic because of Proposition 5. However, instead of computing $\llbracket \text {dead}(U)\rrbracket$ explicitly, there is a more efficient way to express this constraint. Intuitively, $\llbracket \text {dis}(U)\rrbracket = \llbracket \text {dead}(U)\rrbracket$ is the case if enabling a transition $u \in U$ requires to have previously enabled some transition $u' \in U$. This observation leads to:

#### Proposition 10

A set *U* of transitions satisfies $\llbracket \text {dis}(U)\rrbracket = \llbracket \text {dead}(U)\rrbracket$ iff it satisfies the existential Presburger formulawhere  is defined by  for $\textit{\textbf{x}},\textit{\textbf{y}} \in \mathbb {N}^Q$.

This allows us to give the constraint $\textit{lin-layer}(U, \textit{\textbf{a}})$, which is of polynomial size:

$$\begin{aligned} \textit{lin-layer}(U, \textit{\textbf{a}}) {\mathop {=}\limits ^{\scriptscriptstyle \text {def}}}\textit{lin-layer-fun}(U, \textit{\textbf{a}}) \wedge \textit{dis-eq-dead}(U). \end{aligned}$$

### Comparing Ranking and Layer Functions

The ranking and layer functions of Sects. 7.1 and 7.2 are incomparable in power, that is, there are sets of transitions for which a ranking function but no layer function exists, and vice versa. This is shown by the following two systems: 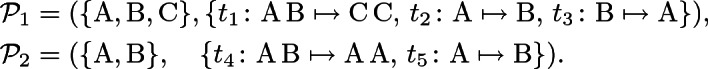


Consider the system $\mathcal {P}_1$, and let $\mathcal {S}= \mathbb {N}^Q$, i.e., $\mathcal {S}$ contains all configurations. Transitions $t_2$ and $t_3$ never become dead at  and can thus never be included in any *U*. Transition $t_1$ eventually becomes dead, as shown by the linear ranking function $r(C) = C(\text {A}) + C(\text {B})$ for $U = \{t_1\}$. But for this *U*, the condition **C2** for layer functions is not satisfied, as  , so $\llbracket \text {dis}(U)\rrbracket \ne \llbracket \text {dead}(U)\rrbracket$. Therefore no layer function exists for this *U*.

Consider now the system $\mathcal {P}_2$, again with $\mathcal {S}= \mathbb {N}^Q$, and let $U = \{t_5\}$. Once $t_5$ is disabled, there is no agent in $\text {A}$, so both $t_4$ and $t_5$ are dead. So $\llbracket \text {dis}(U)\rrbracket = \llbracket \text {dead}(U)\rrbracket$. The linear layer function $\ell (C) = C(\text {A})$ satisfies $\textit{lin-layer-fun}(U, \textit{\textbf{a}})$, showing that *U* eventually becomes dead. As $C \xrightarrow {t_4 t_5} C$ for  , there is no ranking function *r* for this *U*, which would need to satisfy $r(C) < r(C)$.

For our implementation of $\textit{AsDead}(\mathcal {S})$, we therefore combine both approaches. We first compute (in polynomial time) the unique maximal set *U* for which there is a linear ranking function. If this *U* is non-empty, we return it, and otherwise compute a set *U* of maximal size for which there is a linear layer function.

## Experimental Results

**Fig. 2.**
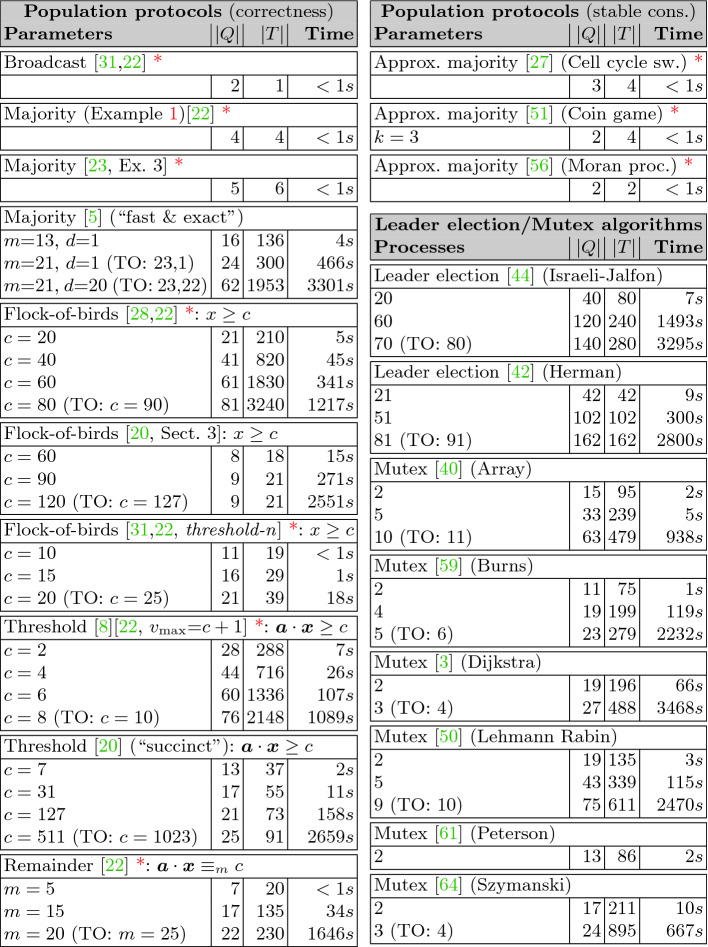
Columns |*Q*|, |*T*|, and **Time** give the number of states and non-silent transitions, and the time for verification. Population protocols are verified for an infinite set of configurations. For parametric families, the smallest instance that could not be verified within one hour is shown in brackets, e.g. (TO: $c = 90$). Leader election and mutex algorithms are verified for one configuration. The number of processes leading to a timeout is given in brackets, e.g. (TO: 10).

We implemented the procedure of Sect. 4 on top of the SMT solver *Z3* [57], and use the Owl [48] and HOA [12] libraries for translating LTL formulas. The resulting tool automatically constructs stage graphs that verify stable termination properties for replicated systems. We evaluated it on two sets of benchmarks, described below. The first set contains population protocols, and the second leader election and mutual exclusion algorithms. All tests where performed on a machine with an Intel Xeon CPU E5-2630 v4 @ 2.20 GHz and 8GB of RAM. The results are depicted in Fig. 2 and can be reproduced by the certified artifact [18]. For parametric families of replicated systems, we always report the largest instance that we were able to verify with a timeout of one hour. For $\textit{IndOverapprox}$, from the approaches in Sect. 5, we use $\textit{IndOverapprox}^0$ in the examples marked with  and $\textit{IndOverapprox}^\infty$ otherwise. Almost all constructed stage graphs are a chain with at most 3 stages. The only exceptions are the stage graphs for the approximate majority protocols that contained a binary split and 5 stages. The size of the Presburger formulas increases with increasing size of the replicated system. In the worst case, this growth can be exponential. However, the growth is linear in all examples marked with  .

*Population Protocols.* Population protocols [8, 9] are replicated systems that compute Presburger predicates following the computation-as-consensus paradigm [10]. Depending on whether the initial configuration of agents satisfies the predicate or not, the agents of a correct protocol eventually agree on the output “yes” or “no”, almost surely. Example 1 can be interpreted as a population protocol for the majority predicate $\text {A}_\text {Y}> \text {A}_\text {N}$, and the two stable termination properties that verify its correctness are described in Example 2. To show that a population protocol correctly computes a given predicate, we thus construct two Presburger stage graphs for the two corresponding stable termination properties. In all these examples, correctness is proved for an infinite set of initial configurations.

Our set of benchmarks contains a broadcast protocol [31], three majority protocols (Example 1, [23, Ex. 3], [5]), and multiple instances of parameterized families of protocols, where each protocol computes a different instance of a parameterized family of predicates^5^. These include various *flock-of-birds* protocol families ([28], [20, Sect. 3], [31, *threshold-n*]) for the family of predicates $x \ge c$ for some constant $c \ge 0$; two families for threshold predicates of the form $\textit{\textbf{a}} \cdot \textit{\textbf{x}} \ge c$ [8, 20]; and one family for remainder protocols of the form $\textit{\textbf{a}} \cdot \textit{\textbf{x}} \equiv _m c$ [22]. Further, we check approximate majority protocols ([27, 56], [51, *coin game*]). As these protocols only compute the predicate with large probability but not almost surely, we only verify that they always converge to a stable consensus.

*Comparison with* [22]. The approach of [22] can only be applied to so-called *strongly-silent* protocols. However, this class does not contain many fast and succinct protocols recently developed for different tasks [4, 17, 20].

We are able to verify all six protocols reported in [22]. Further, we are also able to verify the fast Majority [5] protocol as well as the succinct protocols Flock-of-birds [20, Sect. 3] and Threshold [20]. All three protocols are not strongly-silent. Although our approach is more general and complete, the time to verify many strongly-silent protocol does not differ significantly between the two approaches. Exceptions are the Flock-of-birds [28] protocols where we are faster ( [22] reaches the timeout at $c=55$) as well as the Remainder and the Flock-of-birds-threshold-*n* protocols where we are substantially slower ( [22] reaches the timeout at $m=80$ and $c=350$, respectively). Loosely speaking, the approach of [22] can be faster because they compute inductive overapproximations using an iterative procedure instead of $\textit{PotReach}$. In some instances already a very weak overapproximation, much less precise than $\textit{PotReach}$, suffices to verify the result. Our procedure can be adapted to accommodate this (it essentially amounts to first running the procedure of [22], and if it is inconclusive then run ours).

*Other Distributed Algorithms.* We have also used our approach to verify arbitrary LTL liveness properties of non-parameterized systems with arbitrary communication structure. For this we apply standard automata-theoretic techniques and construct a product of the system and a *limit-deterministic Büchi automaton* for the negation of the property. Checking that no fair runs of the product are accepted by the automaton reduces to checking a stable termination property.

Since we only check correctness of one single finite-state system, we can also apply a probabilistic model checker based on state-space exploration. However, our technique delivers a stage graph, which plays two roles. First, it gives an explanation of why the property holds in terms of invariants and ranking functions, and second, it is a certificate of correctness that can be efficiently checked by independent means.

We verify liveness properties for several leader election and mutex algorithms from the literature [3, 40, 42, 44, 50, 59, 61, 64] under the assumption of a probabilistic scheduler. For the leader election algorithms, we check that a leader is eventually chosen; for the mutex algorithms, we check that the first process enters its critical section infinitely often.

*Comparison with PRISM* [49]. We compared execution times for verification by our technique and by PRISM on the same models. While PRISM only needs a few seconds to verify instances of the mutex algorithms [3, 40, 50, 59, 61, 64] where we reach the time limit, it reaches the memory limit for the two leader election algorithms [42, 44] already for 70 and 71 processes, which we can still verify.

## Conclusion and Further Work

We have presented stage graphs, a sound and complete technique for the verification of stable termination properties of replicated systems, an important class of parameterized systems. Using deep results of the theory of Petri nets, we have shown that Presburger stage graphs, a class of stage graphs whose correctness can be reduced to the satisfiability problem of Presburger arithmetic, are also sound and complete. This provides a decision procedure for the verification of termination properties, which is of theoretical nature since it involves a blind enumeration of candidates for Presburger stage graphs. For this reason, we have presented a technique for the algorithmic construction of Presburger stage graphs, designed to exploit the strengths of SMT-solvers for existential Presburger formulas, i.e., integer linear constraints. Loosely speaking, the technique searches for *linear* functions certifying the progress between stages, even though only the much larger class of Presburger functions guarantees completeness.

We have conducted extensive experiments on a large set of benchmarks. In particular, our approach is able to prove correctness of nearly all the standard protocols described in the literature, including several protocols that could not be proved by the technique of [22], which only worked for so-called strongly-silent protocols. We have also successfully applied the technique to some self-stabilization algorithms, leader election and mutual exclusion algorithms.

Our technique is based on the mechanized search for invariants and ranking functions. It avoids the use of state-space exploration as much as possible. For this reason, it also makes sense as a technique for the verification of liveness properties of non-parameterized systems with a finite but very large state space.

## Footnotes

1 Under the name of *chemical reaction networks*.

2 In the paper, we will omit the silent transitions when giving replicated systems.

3 This follows easily from the fact that $post ^*(\psi )$ is not always expressible in Presburger arithmetic for vector addition systems, even if $\psi$ denotes a single configuration [43].

4 Observe that if $C^\omega (q) = \omega$, then the term “$C(q) \le \omega$” is equivalent to “$\mathbf {true}$”.

5 Notice that for each protocol we check correctness for all inputs; we cannot yet automatically verify that infinitely many protocols are correct, each of them for all possible inputs.

## References

[1] Abdulla PA (2012). Regular model checking. Int. J. Softw. Tools Technol. Transf..

[2] Abdulla, P.A., Cerans, K., Jonsson, B., Tsay, Y.: General decidability theorems for infinite-state systems. In: Proceedings of the 11th Annual IEEE Symposium on Logic in Computer Science, LICS 1996, New Brunswick, New Jersey, USA, 27–30 July 1996, pp. 313–321. IEEE Computer Society (1996). 10.1109/LICS.1996.561359

[3] Abdulla PA, Delzanno G, Henda NB, Rezine A, Grumberg O, Huth M (2007). Regular model checking without transducers (on efficient verification of parameterized systems). Tools and Algorithms for the Construction and Analysis of Systems.

[4] Alistarh D, Gelashvili R (2018). Recent algorithmic advances in population protocols. SIGACT News.

[5] Alistarh, D., Gelashvili, R., Vojnovic, M.: Fast and exact majority in population protocols. In: Georgiou, C., Spirakis, P.G. (eds.) Proceedings of the 34th ACM Symposium on Principles of Distributed Computing, PODC 2015, Donostia-San Sebastián, Spain, 21–23 July 2015, pp. 47–56. ACM (2015). 10.1145/2767386.2767429

[6] Aminof B, Rubin S, Zuleger F, Spegni F, Halldórsson MM, Iwama K, Kobayashi N, Speckmann B (2015). Liveness of parameterized timed networks. Automata, Languages, and Programming.

[7] Angluin D (1987). Learning regular sets from queries and counterexamples. Inf. Comput..

[8] Angluin, D., Aspnes, J., Diamadi, Z., Fischer, M.J., Peralta, R.: Computation in networks of passively mobile finite-state sensors. In: Chaudhuri, S., Kutten, S. (eds.) Proceedings of the 23rd Annual ACM Symposium on Principles of Distributed Computing, PODC 2004, St. John’s, Newfoundland, Canada, 25–28 July 2004, pp. 290–299. ACM (2004). 10.1145/1011767.1011810

[9] Angluin D, Aspnes J, Diamadi Z, Fischer MJ, Peralta R (2006). Computation in networks of passively mobile finite-state sensors. Distrib. Comput..

[10] Angluin D, Aspnes J, Eisenstat D, Ruppert E (2007). The computational power of population protocols. Distrib. Comput..

[11] Athanasiou K, Liu P, Wahl T, Olivetti N, Tiwari A (2016). Unbounded-thread program verification using thread-state equations. Automated Reasoning.

[12] Babiak T, Kroening D, Păsăreanu CS (2015). The Hanoi omega-automata format. Computer Aided Verification.

[13] Baier C, Katoen J (2008). Principles of Model Checking.

[14] Basler G, Mazzucchi M, Wahl T, Kroening D, Bouajjani A, Maler O (2009). Symbolic counter abstraction for concurrent software. Computer Aided Verification.

[15] Berman L (1980). The complexitiy of logical theories. Theoret. Comput. Sci..

[16] Bloem, R., Jacobs, S., Khalimov, A., Konnov, I., Rubin, S., Veith, H., Widder, J.: Decidability of Parameterized Verification. Synthesis Lectures on Distributed Computing Theory. Morgan & Claypool Publishers (2015). 10.2200/S00658ED1V01Y201508DCT013

[17] Blondin, M., Esparza, J., Genest, B., Helfrich, M., Jaax, S.: Succinct population protocols for presburger arithmetic. In: Proceedings of 37th International Symposium on Theoretical Aspects of Computer Science, STACS 2020, 10–13 March 2020, Montpellier, France. LIPIcs, vol. 154, pp. 40:1–40:15. Schloss Dagstuhl - Leibniz-Zentrum für Informatik (2020). 10.4230/LIPIcs.STACS.2020.40

[18] Blondin, M., Esparza, J., Helfrich, M., Kučera, A., Meyer, P.J.: Artifact evaluation VM and instructions to generate experimental results for the CAV20 paper: checking Qualitative Liveness Properties of Replicated Systems with Stochastic Scheduling. figshare:12295982 (2020). 10.6084/m9.figshare.12295982.v2

[19] Blondin, M., Esparza, J., Helfrich, M., Kučera, A., Meyer, P.J.: Checking qualitative liveness properties of replicated systems with stochastic scheduling. arXiv:2005.03555 [cs.LO] (2020). https://arxiv.org/abs/2005.03555

[20] Blondin, M., Esparza, J., Jaax, S.: Large flocks of small birds: on the minimal size of population protocols. In: Proceedings of 35th Symposium on Theoretical Aspects of Computer Science, STACS 2018, 28 February - 3 March 2018, Caen, France. LIPIcs, vol. 96, pp. 16:1–16:14. Schloss Dagstuhl - Leibniz-Zentrum für Informatik (2018). 10.4230/LIPIcs.STACS.2018.16

[21] Blondin M, Esparza J, Jaax S, Chockler H, Weissenbacher G (2018). Peregrine: a tool for the analysis of population protocols. Computer Aided Verification.

[22] Blondin, M., Esparza, J., Jaax, S., Meyer, P.J.: Towards efficient verification of population protocols. In: Schiller, E.M., Schwarzmann, A.A. (eds.) Proceedings of 36th ACM Symposium on Principles of Distributed Computing, PODC 2017, Washington, DC, USA, 25–27 July 2017, pp. 423–430. ACM (2017). 10.1145/3087801.3087816

[23] Blondin, M., Esparza, J., Kučera, A.: Automatic analysis of expected termination time for population protocols. In: Schewe, S., Zhang, L. (eds.) Proceedings of 29th International Conference on Concurrency Theory, CONCUR 2018, 4–7 September 2018, Beijing, China. LIPIcs, vol. 118, pp. 33:1–33:16. Schloss Dagstuhl - Leibniz-Zentrum für Informatik (2018). 10.4230/LIPIcs.CONCUR.2018.33

[24] Blondin M, Finkel A, Haase C, Haddad S (2017). The logical view on continuous petri nets. ACM Trans. Comput. Log. (TOCL).

[25] Bouajjani A, Jonsson B, Nilsson M, Touili T, Emerson EA, Sistla AP (2000). Regular model checking. Computer Aided Verification.

[26] Browne MC, Clarke EM, Grumberg O (1989). Reasoning about networks with many identical finite state processes. Inf. Comput..

[27] Cardelli L, Csikász-Nagy A (2012). The cell cycle switch computes approximate majority. Sci. Rep..

[28] Chatzigiannakis I, Michail O, Spirakis PG, Dolev S, Cobb J, Fischer M, Yung M (2010). Algorithmic verification of population protocols. Stabilization, Safety, and Security of Distributed Systems.

[29] Chen, Y., Hong, C., Lin, A.W., Rümmer, P.: Learning to prove safety over parameterised concurrent systems. In: Stewart, D., Weissenbacher, G. (eds.) Proceedings of 17th International Conference on Formal Methods in Computer Aided Design, FMCAD 2017, Vienna, Austria, 2–6 October 2017, pp. 76–83. IEEE (2017). 10.23919/FMCAD.2017.8102244

[30] Clarke E, Talupur M, Touili T, Veith H, Gardner P, Yoshida N (2004). Verification by network decomposition. CONCUR 2004 - Concurrency Theory.

[31] Clément, J., Delporte-Gallet, C., Fauconnier, H., Sighireanu, M.: Guidelines for the verification of population protocols. In: Proceedings of 31st International Conference on Distributed Computing Systems, ICDCS 2011, Minneapolis, Minnesota, USA, 20–24 June 2011, pp. 215–224. IEEE Computer Society (2011). 10.1109/ICDCS.2011.36

[32] Cooper DC (1972). Theorem proving in arithmetic without multiplication. Mach. Intell..

[33] Czerwinski, W., Lasota, S., Lazic, R., Leroux, J., Mazowiecki, F.: The reachability problem for petri nets is not elementary. In: Charikar, M., Cohen, E. (eds.) Proceedings of 51st Annual ACM SIGACT Symposium on Theory of Computing, STOC 2019, Phoenix, AZ, USA, 23–26 June 2019, pp. 24–33. ACM (2019). 10.1145/3313276.3316369

[34] Emerson EA, Namjoshi KS (2003). On reasoning about rings. Int. J. Found. Comput. Sci..

[35] Esparza, J., Ganty, P., Leroux, J., Majumdar, R.: Model checking population protocols. In: Lal, A., Akshay, S., Saurabh, S., Sen, S. (eds.) Proceedings of 36th IARCS Annual Conference on Foundations of Software Technology and Theoretical Computer Science, FSTTCS 2016, Chennai, India, 13–15 December 2016. LIPIcs, vol. 65, pp. 27:1–27:14. Schloss Dagstuhl - Leibniz-Zentrum für Informatik (2016). 10.4230/LIPIcs.FSTTCS.2016.27

[36] Esparza J, Ganty P, Leroux J, Majumdar R (2017). Verification of population protocols. Acta Inf..

[37] Esparza J, Ledesma-Garza R, Majumdar R, Meyer P, Niksic F, Biere A, Bloem R (2014). An SMT-based approach to coverability analysis. Computer Aided Verification.

[38] Esparza, J., Meyer, P.J.: An SMT-based approach to fair termination analysis. In: Kaivola, R., Wahl, T. (eds.) Proceedings of 15th International Conference on Formal Methods in Computer-Aided Design, FMCAD 2015, Austin, Texas, USA, 27–30 September 2015, pp. 49–56. IEEE (2015)

[39] Finkel A, Schnoebelen P (2001). Well-structured transition systems everywhere!. Theoret. Comput. Sci..

[40] Fribourg, L., Olsén, H.: Reachability sets of parameterized rings as regular languages. In: Moller, F. (ed.) Proceedings of 2nd International Workshop on Verification of Infinite State Systems, Infinity 1997, Bologna, Italy, 11–12 July 1997. Electronic Notes in Theoretical Computer Science, vol. 9, p. 40. Elsevier (1997). 10.1016/S1571-0661(05)80427-X

[41] German SM, Sistla AP (1992). Reasoning about systems with many processes. J. ACM.

[42] Herman T (1990). Probabilistic self-stabilization. Inf. Process. Lett..

[43] Hopcroft JE, Pansiot J (1979). On the reachability problem for 5-dimensional vector addition systems. Theoret. Comput. Sci..

[44] Israeli, A., Jalfon, M.: Token management schemes and random walks yield self-stabilizing mutual exclusion. In: Dwork, C. (ed.) Proceedings of 9th Annual ACM Symposium on Principles of Distributed Computing, PODC 1990, Quebec City, Quebec, Canada, 22–24 August 1990, pp. 119–131. ACM (1990). 10.1145/93385.93409

[45] Jancar P, Purser D (2019). Structural liveness of petri nets is expspace-hard and decidable. Acta Inf..

[46] Kaiser A, Kroening D, Wahl T, Touili T, Cook B, Jackson P (2010). Dynamic cutoff detection in parameterized concurrent programs. Computer Aided Verification.

[47] Kaiser A, Kroening D, Wahl T (2014). A widening approach to multithreaded program verification. ACM Trans. Program. Lang. Syst..

[48] Křetínský J, Meggendorfer T, Sickert S, Lahiri SK, Wang C (2018). Owl: a library for $$\omega $$-words, automata, and LTL. Automated Technology for Verification and Analysis.

[49] Kwiatkowska M, Norman G, Parker D, Gopalakrishnan G, Qadeer S (2011). PRISM 4.0: verification of probabilistic real-time systems. Computer Aided Verification.

[50] Lehmann, D., Rabin, M.O.: On the advantages of free choice: a symmetric and fully distributed solution to the dining philosophers problem. In: White, J., Lipton, R.J., Goldberg, P.C. (eds.) Proceedings of 8th Annual ACM Symposium on Principles of Programming Languages, POPL 1981, Williamsburg, Virginia, USA, January 1981, pp. 133–138. ACM Press (1981). 10.1145/567532.567547

[51] Lengál O, Lin AW, Majumdar R, Rümmer P, Legay A, Margaria T (2017). Fair termination for parameterized probabilistic concurrent systems. Tools and Algorithms for the Construction and Analysis of Systems.

[52] Leroux, J.: Vector addition systems reachability problem (a simpler solution). In: Voronkov, A. (ed.) Proceedings of the Alan Turing Centenary Conference, Turing 100, Manchester, UK, 22–25 June 2012. EPiC Series in Computing, vol. 10, pp. 214–228. EasyChair (2012). 10.29007/bnx2

[53] Leroux, J.: Presburger vector addition systems. In: Proceedings of 28th Annual ACM/IEEE Symposium on Logic in Computer Science, LICS 2013, New Orleans, LA, USA, 25–28 June 2013. pp. 23–32. IEEE Computer Society (2013). 10.1109/LICS.2013.7

[54] Leroux, J.: Vector addition system reversible reachability problem. Log. Methods Comput. Sci. **9**(1) (2013). 10.2168/LMCS-9(1:5)2013

[55] Lin AW, Rümmer P, Chaudhuri S, Farzan A (2016). Liveness of randomised parameterised systems under arbitrary schedulers. Computer Aided Verification.

[56] Moran PAP (1958). Random processes in genetics. Math. Proc. Cambridge Philos. Soc..

[57] de Moura L, Bjørner N, Ramakrishnan CR, Rehof J (2008). Z3: an efficient SMT solver. Tools and Algorithms for the Construction and Analysis of Systems.

[58] Navlakha S, Bar-Joseph Z (2015). Distributed information processing in biological and computational systems. Commun. ACM.

[59] Nilsson, M.: Regular model checking. Ph.D. thesis, Uppsala University (2000)

[60] Pang, J., Luo, Z., Deng, Y.: On automatic verification of self-stabilizing population protocols. In: Proceedings of 2nd IEEE/IFIP International Symposium on Theoretical Aspects of Software Engineering, TASE 2008, 17–19 June 2008, Nanjing, China, pp. 185–192. IEEE Computer Society (2008). 10.1109/TASE.2008.8

[61] Peterson GL (1981). Myths about the mutual exclusion problem. Inf. Process. Lett..

[62] Presburger, M.: Über die Vollständigkeit eines gewissen Systems der Arithmetik ganzer Zahlen, in welchem die Addition als einzige Operation hervortritt. Comptes Rendus du $$\text{I}^\text{ er }$$ Congrès des mathématiciens des pays slaves, pp. 192–201 (1929)

[63] Sun J, Liu Y, Dong JS, Pang J, Bouajjani A, Maler O (2009). PAT: towards flexible verification under fairness. Computer Aided Verification.

[64] Szymanski, B.K.: A simple solution to Lamport’s concurrent programming problem with linear wait. In: Lenfant, J. (ed.) Proceedings of 2nd International Conference on Supercomputing, ICS 1988, Saint Malo, France, 4–8 July 1988, pp. 621–626. ACM (1988). 10.1145/55364.55425

[65] Vardi, M.Y.: Automatic verification of probabilistic concurrent finite-state programs. In: Proceedings of 26th Annual Symposium on Foundations of Computer Science, FOCS 1985, Portland, Oregon, USA, 21–23 October 1985, pp. 327–338. IEEE Computer Society (1985). 10.1109/SFCS.1985.12

